# Hospitalized elder abuse in Iran: a qualitative study

**DOI:** 10.1186/s12877-019-1331-8

**Published:** 2019-11-12

**Authors:** Zeinab Naderi, Sakineh Gholamzadeh, Ladan Zarshenas, Abbas Ebadi

**Affiliations:** 1 Student Research Committee, School of Nursing and Midwifery, Shiraz University of Medical Sciences, Shiraz, Iran; 2 Department of Nursing, Sirjan School of Medical Sciences, Sirjan, Iran; 3 Community Based Psychiatric Care Research Center, School of Nursing and Midwifery, Shiraz University of Medical Sciences, Shiraz, Iran; 4 Behavioral Sciences Research Center, Life Style Institute, Baqiyatallah University of Medical Sciences, Tehran, Iran; 5 Faculty of Nursing, Baqiyatallah University of Medical Sciences, Tehran, Iran

**Keywords:** Elder abuse, Elder neglect, Abuse in health care, Hospitalization, Qualitative study

## Abstract

**Background:**

Elder abuse is a serious violation of human rights and a worldwide issue. Upon hospital admission, elderly patients become vulnerable and susceptible to abuse. Understanding the issues perceived as abuse by the elderly patients and their family members allows us to identify, manage, and prevent elder abuse; especially in hospital settings. The present study aimed to identify and describe the abuse of hospitalized elders from the perspective of patients and their family members.

**Methods:**

The present exploratory qualitative study was conducted from October 2017 to September 2018 at six different teaching hospitals affiliated to Shiraz University of Medical Sciences, Shiraz, Iran. The target population was elderly patients in different wards across various hospitals and their family members. Based on the purposive sampling method, 16 hospitalized elderly patients and 11 family members were recruited and interviewed. The data were analyzed using the inductive content analysis method in accordance with the process described by Elo and Kyngas (J Adv Nurs 62:107–15, 2008).

**Results:**

Based on the analysis of the interview data, four main categories were extracted and classified as Micro-level, Meso-level, Exo-level, and Macro-level issues.

**Conclusion:**

Hospitalized elder abuse is a multi-dimensional phenomenon caused by personal and professional factors as well as issues related to the inadequate physical environment and organizational structure. To prevent the occurrence of elderly abuse, it is recommended to train hospital staff, rearrange the physical environment, reform the organizational structure, and better plan and manage the financial, physical, and human resources.

## Background

Elder abuse is universally recognized as a widespread and serious issue. Due to the global rise in the elderly population, it is anticipated that more elderly will experience abuse in the near future [1]. Compared to other types of interpersonal violence, studies on elder abuse are scarce and still in early stages of research; particularly institutional abuse [2]. Without an overall consensus, different definitions of institutional elder abuse have been provided by various researchers, the majority of which has been subjective and only represented the views of the personnel [3]. Compared to other forms of elderly abuse, institutional abuse has attracted little attention in the literature due to its complexity and difficulty of establishing a proper definition [4]. As stated in a previous study [5], we also felt the need for a broad and flexible definition of institutional elder abuse that covers different forms of abuse and the context in which it may occur. Since current studies on elderly abuse have mainly focused on domestic abuse and consequently there is insufficient information about the dominant factors and characteristics that contribute to elder abuse in institutional settings. The associated facts on the actual prevalence, risk factors, outcomes, and the optimal management of institutional elder abuse are not readily available [6]. It has been reported that the rate of institutional elder abuse is much higher than at the community level [2, 7]. The statistics obtained through reports by the staff at institutions have indicated elder abuse at an overall prevalence rate of 64.2%. Elderly self-report declarations have shown that the highest and lowest rates were associated with psychological abuse (33.4%) and sexual abuse (1.9%), respectively [2, 8].

Typical institutions where elder abuse occurs are nursing homes, residential care facilities, hospitals, daycare centers, and even in those centers claiming to offer high-quality care. The scope of abuse and neglect in these institutions is the results of many factors such as the poor quality of provided care, staff shortages, poor facilities, and impractical organizational policies [9]. It is a well-known fact that elderly patients have the highest rate of hospital admissions and often remain in the hospital longer than any other age group [10], which in turn increases the risk of abuse. Furthermore, they are generally more vulnerable as they have less control over their lives and health, and are exposed to the ageist attitude of health care providers; all of which makes them prone to abuse during the period of care [11]. Irrespective of the type of abuse, the elderly may unnecessarily be exposed to suffering, hurt or pain, deprivation of human rights, and a diminished quality of life [9]. As a direct result, they tend to lose faith and confidence in the health care system and may even refuse or postpone hospitalization [12].

The perception of elder abuse in institutions is to a large extent formed by the reports prepared by the health care personnel [13]. Even though information obtained directly from the elderly is essential to define, identify, and manage interventions for the prevention of abuse, only a few studies have included and reported their viewpoints [14]. Published studies on elder abuse have indicated a clear difference between the experiences of the elderly and those presented by the health care professionals [15]. Consequently, it was deemed necessary to determine the perception of the patients themselves on what constituted abuse and what was perceived as offensive, humiliating, impertinent, or abusive [16]. Considering the scarcity of such information [17] and the complex nature of elder abuse at institutions, it was essential to approach the topic from different angles. To do so, the perspective and experience of both the elderly and their family members were included. Family members are the ideal proxies for providing the required information since some elderly patients with cognitive disorders, who are more likely to be abused but generally studied less, might not have the capacity to provide in-depth information [3].

We trust that the findings of the present study would broaden the perception of what abusive behavior constitutes and increase the awareness of not only the elders in need of support, but also of their family members and the treatment team.

## Methods

The study was conducted in accordance with the consolidated criteria for reporting qualitative research (COREQ) [18].

### Objective

The present study aimed to identify and describe the abuse of hospitalized elders from the perspective of elderly patients and their family members.

### Participants and setting

The present exploratory qualitative study was conducted from October 2017 to September 2018 at six different teaching hospitals affiliated to Shiraz University of Medical Sciences, Shiraz, Iran. The target population was elderly patients in different wards (internal medicine, surgery, emergency, CCU, ICU) across various hospitals as well as their family members.

The purposive sampling method was used to recruit the participants. The sampling continued until data saturation (i.e., no new substantive data or new category was acquired). Selection of the participants was based on the variation of samples and the progression of the interviews. The inclusion criteria for elderly patients were: aged ≥60 years, current hospitalization for more than 3 days, physical/mental/psychological ability to participate in an interview, willingness to participate, capacity to provide in-depth information, and adequate verbal communication skills. In addition, the inclusion criteria for the family members were: aged ≥18 years, having accompanied the patient for a minimum of 3 days during the current hospitalization, capacity to provide in-depth information, physical/mental/psychological ability to participate in an interview, and willingness to participate. Accordingly, 16 hospitalized elders and 11 family members were enrolled in the study.

In the absence of any specific protocol in Iran to identify institutional elder abuse (e.g., in hospitals), a dedicated approach was used to identify suitable participants. Based on the experience and informed judgment of the first author/interviewer, and in accordance with the inclusion criteria, potential participants were approached in various hospitals and at different hospital work shifts. Only those who had adequate knowledge about and experience with elder abuse were recruited. Initially, they were informed about the research goals and method, and the non-commercial origin of the sponsor was described. In addition, the confidentiality of any disclosed information was guaranteed and voluntary participation was emphasized. Subsequently, written informed consent was obtained from all the participants. Demographic characteristics of the participants included age, sex, level of education, the length of stay in the hospital, and the type of hospital ward (Table 1).

**Table 1:** Clinical and demographic characteristics of the participants

Participant	Age range (years)	Sex	Education Level	Ward	LOS (days)
P1	60–70	Female	Diploma	Surgery	4
P2	70–80	Female	Illiterate	Surgery	7
P3	60–70	Male	Secondary school	Medical	9
P4	70–80	Male	Illiterate	Surgery	10
P5	60–70	Female	Diploma	Surgery	21
P6	70–80	Male	Secondary school	Medical	15
PFM7	50–60	Female	Higher diploma	ICU	21
P8	70–80	Male	Secondary school	CCU	5
PFM9	30–40	Female	Diploma	Surgery	11
P10	70–80	Male	Secondary school	Medical	24
PFM11	40–50	Female	Bachelor’s degree	Surgery	17
PFM12	30–40	Female	Higher diploma	Surgery	9
P13	60–70	Male	Higher diploma	Medical	9
PFM14	40–50	Male	Diploma	Emergency	6
P15	80–90	Male	Elementary school	Surgery	12
P16	60–70	Female	Higher diploma	Surgery	14
PFM17	30–40	Female	Master’s degree	Surgery	13
P18	60–70	Female	Elementary school	Medical	7
PFM19	50–60	Female	Secondary school	Medical	13
PFM20	30–40	Male	Bachelor’s degree	Surgery	11
PFM21	20–30	Male	Master’s degree	Medical	8
PFM22	20–30	Female	Bachelor’s degree	Medical	22
P23	80–90	Male	Elementary school	Medical	6
P24	70–80	Male	Elementary school	Emergency	4
PFM25	20–30	Female	Diploma	Medical	20
P26	70–80	Female	Elementary school	Medical	16
P27	60–70	Female	Bachelor degree	Surgery	8

*P* Patient, *PFM* Patients’ family member, *LOS* Length of stay in hospital, *CCU* Coronary care unit, *ICU* Intensive care unit

### Data collection

The data were collected through 29 individual in-depth semi-structured interviews, as recommended in a previous study [19]. Note that two participants were interviewed twice in order to clarify ambiguities and obtain complementary information. The face-to-face interviews were conducted by the first author (ZN) and lasted between 35 to 75 min.

Following prior coordination and approval from the hospital authorities, the interviews were held in a private hospital room, where only the participant and the interviewer were present. The participants were informed about the possibility of an additional interview if further information was required. With the permission of the participants, an audio recording of the interviews was made and subsequently transcribed verbatim. Additionally, in support of data analysis, field notes during and after each interview were made by the interviewer. Further enhancement was achieved through a voluntary review of the transcription by some of the literate participants.

In order to focus on the characteristics and dimensions of hospitalized elder abuse, a pilot interview was conducted with a patient and a family member (not the actual participants); based on which the final version of the interview guide was formulated (Table 2). The interview guide mainly focused on perceptions, attitudes, and definitions related to hospitalized elder abuse from the perspective of the participants, as well as their current/past experiences. The interviews started with a series of general questions followed by detailed questions to clarify the responses and to extract complementary information.

**Table 2:** Semi-structured interview guide for elderly patients and family members

Main questions	Probing questions
List for elderly patients	
What is your understanding of the term abuse of hospitalized elders and what does it mean to you?	Please give some examples of what you consider as abusive behavior.
What is the first thing that comes to your mind when the term abuse of hospitalized elders is mentioned?	
Have you experienced abuse by a nurse, doctor, or other health care personnel during the current hospital setting?	Please expand. What happened? How did you react? How did you feel? When did you notice being abused? Do you think it was done intentionally to harm you?
Do you have previous experience of abuse by health care personnel while you were hospitalized?	
Have you ever witnessed the abuse of other elderly patients by health care personnel?	Please describe the situation in details?
Is there anything else you would like to add to this topic?	Please give an example.
List for family members	
What is your understanding of the term abuse of hospitalized elders and what does it mean to you?	Please give some examples of what you consider as abusive behavior.
What is the first thing that comes to your mind when the term abuse of hospitalized elders is mentioned?	
Have you ever noticed your elderly family member being abused by health care personnel during the current hospitalization?	Please expand. What happened? When did you notice that your elderly patient was abused? Do you think it was done intentionally to harm your patient?
Do you have previous experience of abuse of a hospitalized elderly family member by health care personnel?	
Have you ever witnessed other elderly patients being abused by a health care provider?	Please describe the situation in details?
Is there anything else you would like to add to this topic?	Please give an example.

### Data analysis

The data were analyzed using the inductive content analysis method in accordance with the process described by Elo and Kyngas [20]. This method was specifically chosen since there were no previous studies on hospitalized elder abuse and the currently available information was fragmented [20]. The data were analyzed in three stages, namely preparation, organization, and reporting of the results. The preparation stage included a review of the transcriptions as the main source of information to analyze and establish a logical link between the data and the main topic (through immediate transcription of the interviews upon completion, followed by a repeated study of the text to gain in-depth understanding). The organization stage included (i) selection of semantic units and open codes, (ii) grouping of the related codes, (iii) categorization of similar groups, and (iv) the abstraction process to form main categories. The final stage involved reporting the extracted categories. Note that the extracted categories evolved after analyzing the data from each interview until data saturation. Data organization and management was performed using MAXQDA 2007 software.

After each interview, initial data analysis and extraction of codes were carried out by the first and second authors. The role of the first author was to obtain an in-depth understanding of the subject through a repeated study of the transcripts. Subsequently, the initial semantic units and open codes were extracted. Then, the second author reviewed and validated both the transcriptions and the semantics. Possible incompatibilities were resolved in a joint meeting. Based on their conceptual similarity, the extracted codes were classified into sub-categories. The conceptually related sub-categories were then grouped into generic categories, and these were finally grouped into main categories. The above-mentioned process was a joint effort by the entire research team and the final list of sub-categories, generic categories, and main categories was formulated after many discussions and multiple joint meetings.

### Rigor

Data trustworthiness was assessed using the four criteria proposed by Lincoln and Guba [21]. The credibility and confirmability criteria were achieved through prolonged engagement with the research (11 months) to ensure in-depth understanding of the subject, prevent potential deviations, avoid the collection of incorrect information, and confirm data saturation while formulating the main categories. Specific precautions were taken and adequate time was allocated to ensure extensive data collection (physical presence in the field for purposive sampling, selecting and recruiting participants to ensure maximum diversity), data analysis in between every interview (meticulous verbatim transcription of the audio files, repeated review of the transcripts, grouping of the field notes, classification of the extracted codes and categories); cross-checking transcripts with some of the participants to ensure accuracy, an iterative process between data and analysis process over a relatively long period of time, and finally repetition of two interviews because of ambiguities. Furthermore, an audit trail was maintained as the study progressed and the results were confirmed through external checks. In addition, to achieve credibility and confirmability, triangulation of the participants (elderly patients and their family members), time (different hospital work shifts), space (different wards at six hospitals), member checking, and peer debriefing and review was implemented.

To ensure confirmability and dependability of data, the audit trail was supported by different types of documents, including raw data (participants’ quotes), results of data analysis and data reduction (main categories, generic categories, sub-categories, and codes), progress report (methodology), documents related to the goals and researcher’s attitude (reflexive notes on researcher’s assumptions), and finally the results.

To improve the level of transferability, information such as demographic characteristics, study conditions, interview techniques, data collection method, and data analysis process were adequately described. In addition, a detailed description of the categories, presentation of verbatim quotations from the participants, identification of diverse cases, and purposive sampling were used [22, 23].

## Results

A total of 27 individuals participated in the study of which 16 were elderly patients and 11 family members. The patients and family members were aged 60–83 years and 23–55 years, respectively (Table 1). Analysis of the interview data resulted in 1313 codes, 31 sub-categories, 10 generic categories, and 4 main categories (Table 3). The main categories were classified as Micro-level, Meso-level, Exo-level, and Macro-level issues.

**Table 3:** Main categories, generic categories, and sub-categories of the abuse of hospitalized elders

Main category	Generic category	Sub-category
Micro-level issues	Physical abuse	Physical aggression/ Rough care and treatment/ Intentional-unintentional bodily harm
	Emotional and psychological abuse	Verbal abuse/ Violation of the patient’s dignity/ Discrimination in the provision of health care services/ Psychological violence/ Deprivation and unnecessary restrictions
Meso-level issues	Insufficient professional competence	Clinical incompetence/ Inadequate psychological empowerment/ Insufficient professional belonging
	Professional negligence	Inadequate emotional support/ Insufficient information support/ Caring neglect/ Medical neglect
	Insufficient adherence to professional ethics	Unethical behavior/ Irresponsibility/ Inappropriate disclosure of facts/ Using elderly patients as teaching tools
Exo-level issues	Unsanitary environment	Unhygienic conditions/ Failure to adhere to the infection control principles
	Unsafe environment	Inappropriate physical environment/ Confusing conditions
Macro-level issues	Complex process of admission till discharge	Difficult processes of patient’s admission and transfer/ lengthy waiting time/ lengthy process of patient discharge
	Limitations of resources	Insufficient allocation of human resources/ Inadequate facilities and equipment
	Financial abuse	Financial neglect/ Imposing extra costs on patient/ Patient’s treatment to achieve more income

### Micro-level issues

This main category included the violation of hospitalized elderly patients’ integrity at the individual/personal level. It included two generic categories, namely “Physical abuse” and “Emotional and psychological abuse”.

#### Physical abuse

Physical aggression, as a sub-category of physical abuse, was depicted by the participants as rare skirmishes with the hospital staff and included being beaten, pushed, and thrown to the ground. Such aggressive behavior was mainly displayed by the hospital security staff against patients and family members. The sub-category “Rough care and treatment” included inappropriate actions by the hospital staff such as unnecessary use of physical restraints, rough handling of elderly patients, sudden repositioning of patients immediately after surgery, abrupt and painful removal of adhesive tapes and dressings, or rough wound cleaning. Other examples of rough care and treatment were rough behavior when waking a patient to administer drugs, during bathing, changing clothes/adult diapers, patient handling, catheter replacement, restraint for physical examination, or venipuncture. A female patient stated: “*I am extremely dissatisfied with the provided nursing care. They caused me a lot of unnecessary pain by abruptly removing my wound dressing. It was as if they were angry about something.*” [P2].

Similarly, a female family member stated: “*They treated my mother as a prisoner. They chained her hands to the hospital bed to prevent catheter removal and put an oxygen mask with a large reservoir bag on her face. It was a distressful scene since the restraint was to the extent that she could not move her wrists or reposition her body to relieve the pain caused by bedsores. To calm her down, I tried to hold her hand. She initially refused due to extreme tiredness and fear, but then she held my hand tight and shed tears.*” [PFM11].

The sub-category “Intentional/unintentional bodily harm” included physical harm to elderly patients during relocation within the hospital, injuries due to a fall, excessive and repetitive clinical examination and treatment, injuries due to rough nursing care; and injuries as a result of teaching medical and nursing students to identify the appropriate veins to puncture, arterial blood sampling, and other invasive procedures. Injuries of this kind combined with the development of hospital-acquired pressure ulcers and nosocomial infections constituted a violation of patients’ physical integrity. A female family member stated: “*I noticed a 90-year-old obese patient with a high blood sugar level on the emergency ward. He was hospitalized on a stretcher rather than in a proper bed. Consequently, he developed pressure ulcers within 3 days.*” [PFM12].

#### Emotional and psychological abuse

This generic category covered other manifestations of micro-level issues. It included verbal and psychological violence, such as displaying aggressiveness toward the elderly patient because of their slow walking speed or whimpering due to pain; yelling at them for being irritating, being uncooperative, and refusing clinical examination, venipuncture, and other diagnostic procedures. Moreover, verbal abuse and the use of inappropriate language, disrespectful treatment and humiliation of the elderly, accusing an elderly of lying, blaming them for their incontinence, complaints and repeated use of the emergency button were further examples of such abuses. A female family member stated: “*They yelled at my husband, insulted him, and accused him of whining and making too much noise causing discomfort to other patients. My husband was deeply upset and felt humiliated”.* [PFM7].

Some of the participants mentioned direct violations of human dignity by the medical team, which made them feel undervalued. Examples of such behaviors were ignoring a patient during daily rounds, treating patients like objects, and causing them embarrassment because of their incontinence. A female patient stated: “*The treatment team continually disrespected us. It was as if we were not humans; we do not deserve such treatments. I felt it was better to die than experience such humiliations, pain, or having to beg for care.*” [P5].

Undermining patient autonomy was another form of violation of patients’ dignity. It included behaviors such as belittling attitude during treatment and care, ignoring the patients’ preferred choice of treatment or specific treatment team, changing patients’ room or bed without prior notice, or forcing them to blindly follow instructions from the treatment team. A male patient stated: “*We have been totally ignored throughout the treatment process. A patient needs to feel valued by being involved. The physicians should clarify the diagnosis and explain the results in simple terms and obtain patient’s support for the next steps of the treatment. By doing so, a patient regains confidence and peace of mind.*” [P8].

Inadequate verbal and non-verbal communication also undermined the patients’ dignity. The treatment team should engage in an open discussion, listen, and spend adequate time with elderly patients. Instead, they tend to avoid eye contact and prefer discussing matters with their family members. Moreover, they manifest an arrogant attitude toward patients and communicate without any consideration for their visual or audible deficiencies. A male patient stated: “*It was really unacceptable that the doctor simply prevented me from asking a question about my foot disease. When I persisted, he ignored me and said something to the nurse in Latin which was incomprehensible to me.*” [P3].

Invasion of physical privacy was another example of undermining the patients’ dignity. Intrusions were typically in the form of unnecessary exposure of the body parts during clinical examination or care, ignoring the elderly’s cultural tendencies and religious beliefs in terms of clothing, and examination of private parts by the opposite gender. A female family member stated: “*My mother was completely naked when they brought her out of the operating room; only her breasts were covered. Culturally, the older generation is more sensitive about such things and my mother would definitely have considered this an intrusion and an insult.*” [PFM12].

Violation of patients’ privacy extended to the breach of privacy in a hospital ward. Placing hospital beds close to each other or leaving a patient on a stretcher in a corridor without a privacy screen were typical examples of such violations. Breach of patient confidentiality (e.g., being overheard by other patients while a patient’s medical history was taken) was considered a violation of patient’s informational privacy.

Discriminatory provision of health care services and ageism were additional problematic issues, which included age, race, and social status bias against patients. The participants believed that the ageist attitude of staff toward elderly patients (considering them as wicked, labor-intensive, frail, filthy, smelly, and care for the elderly patients as pointless in general) undermined the quality of the provided treatment and care. A male patient stated: “*Some of the personnel had such an attitude. They made comments like this patient is old, too ill, suffers from incontinence, and our lives will become miserable if we admit this patient to our ward. It is best not to admit such patients as they tend to yell. They have lived their lives and are problematic patients.*” [P13].

Unnecessary restrictions on elderly patients such as preventing or shortening of hospital visiting hours for family and friends were also perceived as emotional and psychological abuse.

### Meso-level issues

This main category was associated with the relationship level or characteristics of the perpetrator and the lack of training of health care professionals. It included three generic categories, namely “Insufficient professional competence”, “Professional negligence”, and “Insufficient adherence to professional ethics”.

#### Insufficient professional competence

Clinical incompetence, as a sub-category of insufficient professional competence, was related to the expected level of knowledge and skills to perform common medical procedures such as primary care, examinations, and diagnostic procedures. The participants noted that the treatment team, due to their incompetence, had to repeat procedures. Other observations were poor medical decision-making and even disinterest to acquire the minimum level of competence. Such incompetence could have serious physical and psychological consequences for elderly patients. Therefore, the participants viewed this as unintentional negligence and abuse. A male family member stated: “*Care for and treatment of elderly patients is a delicate matter. Apparently, the treatment team lacked the required specialized behavioral training on how to approach them. In general, it is not unusual for a novice nurse to need multiple attempts to successfully draw blood, but this is unacceptable when dealing with older patients; like my father.*” [PFM21].

The sub-category “Inadequate psychological empowerment” included the lack of self-confidence while caring for and treating the elderly, as well as irritable behavior towards them. Typical examples were impatience while providing care (assistance with a short walk on the ward, eating meals, bathing, dressing, etc.) and in responding to repetitive questions by elderly patients. A male patient stated: “*A nurse should obviously possess a high level of patience when dealing with elderly patients. Particularly, novice nurses tend to lose their temper and perform their duty with reluctance while displaying inappropriate behavior. Physicians are no exception, they let you wait without giving any reason and are generally oversensitive.*” [P13].

A lack of sense of professional belonging was another sub-category of insufficient professional competence. The participants experienced the feeling of being undervalued since the medical staff provided care without any motivation or enthusiasm.

#### Professional negligence

A sub-category associated with such negligence was “Inadequate emotional support”. The participants perceived the absence of emotional support as very disappointing and examples included the display of the bare minimum of empathy and sympathy, indifference to their suffering, and a lack of compassion bordering to cruelty. A female patient stated: “*It is like a factory here and the nurses behave like robots. They arrive on time, give us the medications, say the routine things, and then leave us again. They display no emotions or affection.*” [P16].

Inadequate provision of medical information by the treatment team was also considered as professional negligence. The participants described examples such as ignoring their questions, withholding medical information resulting in uncertainty, inadequate self-care training, and inadequate information about the hospital discharge process. A male patient stated: “*No one properly responds to our questions. The attending physician referred us to the resident doctor and she referred us to the nurse; eventually, the nurse claimed that she was not aware of the given instructions. Even worse, they tend to inform our family members instead of us. At least one person should be responsible for the overall information.*” [P8].

Caring neglect was another sub-category of professional negligence. The participants described various examples of failure and reluctance in providing timely care to the elderly, e.g., delays in attending to a patient, in giving painkillers, changing wound dressing and diapers, and helping with personal hygiene, providing a bedpan, assistance to use the lavatory, and mealtimes. A female family member stated: “*When I visited my mother in the hospital, it was shocking to see the state she was in. They did not change her diaper on time and her bedsore was infected due to exposure to feces. It took the nurses a long time to finally attend to her and when they did, they forgot to disinfect the wound with bedsore cream. Again, it took a while before they returned to attend to the wound.*” [PFM19].

The negligence also extended to poor-quality treatment provided by attending physicians (failure to recognize or timely and appropriately respond to the medical and therapeutic needs of elderly patients), which could be considered medical neglect. Typical examples were the physicians’ diagnostic failure caused by the lack of accuracy, hastiness and delay, or repetition of procedures due to diagnostic errors. Other examples included unnecessary treatments causing a prolonged hospitalization, arbitrary cancelation of surgeries, the immediate association of an illness with older age without any examination, or untimely discharge of an elderly patient. Obviously, these situations could all have serious medical consequences for elderly patients (e.g., the progression of the disease, extra complications, endangering the life of a patient, the risk of maiming and chronic pain, etc.). A male patient stated: “*Due to the repeated cancellation of my surgery, I have not eaten properly during the past 5 days. Every time I get ready for the surgery, I am told it has been canceled and that I can eat. Then, 12 hours later, they instruct me not to eat as the surgery is planned for the following day. They are so disorganized and there is nothing I can do about it.*” [P10].

#### Insufficient adherence to professional ethics

This generic category was associated with the professional behavior of the treatment team. The participants characterized such behavior as irresponsible and unethical, which included inappropriate disclosure of facts, concealment of medical mistakes, and the use of elderly patients as teaching material. A female family member stated: “*It was shocking news to hear that my father’s leg had to be amputated. However, the physician was indifferent about it and disclosed the information as if it was unimportant and routine.*” [PFM17].

### Exo-level issues

This main category was related to the environmental setting in which the abuse had occurred. It included two generic categories, namely “Unsanitary environment” and “Unsafe environment”.

#### Unsanitary environment

This generic category was associated with unhygienic conditions and failure to adhere to basic infection control principles. The participants stated their dissatisfaction with the unhygienic state of the hospital rooms, sanitation, medical instruments, and ward facilities. Moreover, lack of proper air-conditioning, unpleasant odor, and the presence of insects in hospital rooms were mentioned. The treatment team did not adhere to the basic infection control principles. They did not wash their hands, nor used new disposable gloves for the examination of each patient, infectious wastes were not timely disposed of, nor were the bed-linens changed on a regular basis. Such unhealthy conditions could aggravate or further complicate (early onset of hospital-acquired infection) the health of elderly patients. A female family member stated: “*For the records, I took photos of the state of the hospital beds. The mattress was so old, dirty, and uncomfortable. Worse, it was simply covered with a disposable sheet. Our elderly patient suffered from an infectious disease and it was ironic that they offered her a bed in such a condition.*” [PFM22].

#### Unsafe environment

The sub-categories associated with the unsafe environment were inappropriate physical environment and confusing hospital conditions. The participants noted items such as inadequate lighting (especially at night), excessive noise, inadequate air-conditioning, non-adjustable beds, lack of grab rails in toilets and corridors, inadequate signposting, and problems related to the intra-hospital routing of patients. On the same note, additional issues were limited physical space in hospital rooms, the admittance of patients of different ages with different diseases in the same room/ward, allowing insufficient time to get acquainted with the environment and routines, chaotic environment, and irregularity in and uncertainty about treatment and care. A female patient stated: “*The emergency unit looked like a war zone. It was crowded, no place to sit down, chaotic, and filthy. All sorts of patients were there, from young to old, women and men, and they all had different illnesses. I did not know who to go to and simply wished someone would rescue me.*” [P18].

### Macro-level issues

This main category included organizational policies and structural issues organizational malpractice that contributed to elderly abuse. A poorly managed hospital, both in operational and accounting terms, introduced extra challenges to the treatment and care of the elderly patients. While such administrative and organizational barriers can affect the quality of treatment of all hospitalized patients, however, the elderly are more likely to feel neglected due to their greater vulnerability, complex and multiple needs, frequent hospitalization, as well as physical and mental limitations to commute or wait in queues.

#### Complex process of admission till discharge

The participants considered operational shortcomings as the main reason for elderly patients to experience a sense of fatigue, confusion, lost hope, and delayed treatment. Typical examples were the failure to admit or delay in the admission of the elderly patient, a long waiting list, extended length of stay in the emergency unit (up to 1 month) or general ward, incorrect prioritization of ward transfers, and cumbersome decision-making process for hospital discharge. These could cause unnecessary delays in the treatment of elderly patients and result in irreversible physical, mental, and psychological complications such as exacerbation of the illness, mental confusion, and inability to perform daily tasks. A female family member stated: “*I do understand that they are too busy and the hospital is overcrowded, but they should not keep an elderly patient in the emergency unit for six long days prior to surgery. This is a typical example of abuse. Strangely enough, after the surgery, they planned to take our patient with an infectious disease back to the emergency unit due to the unavailability of a free bed on the ward.*” [PFM9].

#### Limitations of resources

Staff shortage, particularly physicians and nurses, could have a significant and negative impact both on the elderly patients and on the available personnel. On the one hand, elderly patients perceived the lack of staff as a sign of negligence in providing care and disrespect of their dignity. While on the other hand, overworked personnel are prone to a higher risk of making unintentional mistakes due to fatigue, burnout, irritability, dissatisfaction, and time shortage. As a direct result, overworked personnel displayed aggressive behavior and the elderly patients received a lower quality of care. A female family member stated: “*My husband was in desperate need of physiotherapy. They claimed a shortage of staff, physicians, and physiotherapist. But time was of the essence and we had already lost a few precious days. Nobody seemed to care.*” [PFM7].

Lack of supplies (e.g., essential drugs, advanced wound care dressings), outdated medical instruments, inappropriate hospital beds and accessories (mattress, blanket, bed- and night linen) were additional conditions that negatively affected the elderly patients.

#### Financial abuse

Financial abuse was defined as ignoring the financial capacity of elderly patients to cover hospitalization costs as well as the provision of care for the sole purpose of achieving the organizational financial target. The participants expressed their dissatisfaction with wide-ranging unexplained expenses during their hospital stay. For instance, they complained about personnel’s negligence in safeguarding their small valuable items by not providing a safety deposit box, which led to the assumption that they were equally as careless in their medical care. In addition, they noted unnecessary expenses due to long-term hospitalization, delayed treatment, medical mistakes, and patient’s obligation to purchase medications. Other financial issues were related to needless referrals to private clinics, unnecessary and ineffective surgeries, paying the physician’s fee separately while it is already included in the hospital invoice, informal payments, and favoritism (though rare). A female patient stated: “*Why do we have to pay chaperone fees as in hotels. Some patients do need the presence of a family member or a friend in such desperate times. What if a patient has no money while in need of someone even for a short while? I’d rather not pay the extra costs despite the need for assistance to get out of bed.*” [P26].

Overall, the results showed that the abuse of hospitalized elderly patients occurred at Micro, Meso, Exo, and Macro levels. The abuse included physical and emotional abuse at the personal level, the neglect of both patients and professional duties, unethical behavior as well as the presence of an unsafe environment and confusing conditions for elderly patients. On top of these issues, shortcomings due to a failing organizational structure, management, and policies (e.g. the cumbersome process from admission till discharge, limitation of financial resources, financial abuse) negatively affected the treatment of elderly patients.

## Discussion

The findings of the present study indicated that micro-level issues that included “Physical abuse” and “Emotional and psychological abuse” were the most important categories of the abuse of hospitalized elderly patients. Verbal and physical abuse of patients in the health care environment is a common occurrence [24]. A previous study stated that the abuse of physical integrity of elderly patients was related to physical abuse and lack of individualized care [25]. A qualitative phenomenological study also reported that physical and psychological abuses were typical examples of the abuse of the elderly in residential settings [26]. In line with our results, other studies have also shown that the privacy, identity, dignity, and autonomy of elderly patients at private institutions and public health care centers were regularly neglected [13, 27]. A study on the violation of ethical principles in institutional care reported that the dignity of elderly patients was violated through infantilization and undignified verbal abuse [28]. Elaine Cass [29] stated in her study that inappropriate behavior, ageism, inequality, discrimination, deprivation, and unfavorable conditions threaten the dignity of elderly patients. She reported that the elderly patients believed they were not listened to and treated as a medical case rather than an individual [29]. In line with our findings, a previous study concluded that ageism among health care providers is widespread, and at times ends up in withholding the necessary treatment from the elderly patients [30].

Neglect in the health care system is a common occurrence and the most distressing aspect of abuse [17, 31]. Our results showed that the neglect of hospitalized elderly by the medical staff was a common form of abuse. According to a previous study, neglect can be as serious as physical abuse and could have a profound effect on one’s health and quality of life [32]. There is a growing concern about the widespread neglect of patients in hospitals [31]. Depriving elderly patients of medical examination is a form of neglect and often occurs when symptoms can be readily attributed to old age without proper diagnosis [33]. Physicians even seem to hesitate in prescribing life-saving intervention and supportive care simply because a patient is too old. Previous studies have reported that many physicians, while providing the necessary information, take less time for older patients [34]. When patients are not provided with any information or when actions are taken without their prior consent, they perceive it as abuse [12]. A previous study reported that failure to provide patients with information is a form of neglect [35]. Neglect of the elderly patients can also be in the form of being ignored or their case being put at a lower care priority; leading to their marginalization [10].

Additional features of negligence by health care providers in performing their professional tasks were negligence in providing care and inadequate emotional support to elderly patients. A previous study stated that the lack of compassion, empathy, and attention of staff for patients is perceived as physical and emotional neglect [36]. Similarly, a study reported that insensitivity, lack of consideration, and indifference to the patient’s feelings and condition were the most common form of abuse in health care [35]. The negative attitudes of nurses towards older patients in the acute hospital setting resulted in performing routine tasks only (observations, medication, technical procedures) and paying less attention to their care needs (nutrition, sanitation, excretion, mobility, and coaching) [10]. Elderly patients are often subjected to inequality in care [37] since health care professionals tend to ignore them due to their physical limitations, reduced autonomy, and repetitive illnesses [34]. However, neglect of the elderly could also be unintentional due to the lack of personal and social skills, [33] incompetence, or carelessness in performing professional duties [36]. According to the literature, most medical personnel have not received the necessary specialized training to provide optimum care to elderly patients and to address their specific needs. It has been shown that trained and experienced personnel are more compassionate toward elderly patients and are more aware of patients’ dignity and self-esteem [27]. Therefore, we recommend that such training should be made mandatory for all personnel at all health care centers.

The results of the present study showed that one of the most important aspects of meso-level issues was the absence of adherence to professional ethics. Professional ethics is a code of conduct that must be adhered to while interacting with patients [38]. Ethical values such as respect for patients’ rights and virtue ethics are the prerequisites for professionalism [39]. A study reported that ethical values have been regularly violated by the personnel in various institutions. Neglecting ethical values could lead to institutional abuse as a norm while the staff might not be even aware of their conduct [28].

Exo-level issues formed another main category of elder abuse in hospitals and were related to the environmental context in which the abuse had occurred. According to the WHO report, health care centers that are crowded, dirty, and noisy can be very confusing places for the elderly. Moreover, unfamiliarity with the environment and medical procedures could negatively affect their usual character and behavior [11]. Unfortunately, most hospitals and acute care centers are not specifically designed to adequately accommodate the needs of the elderly to the extent that they can even be labeled as unsafe and age-unfriendly [40, 41]. In addition, elderly patients are exposed to various medical routines and procedures that are in stark contrast to their uneventful lives. These issues could force them to become more dependent on others, exacerbate their functional impairment, and even cause delirium [42].

Another main aspect of hospitalized elder abuse consisted of macro-level issues related to organizational policies, mismanagement, and structural problems; leading to an experience of abuse and neglect during the treatment process. According to the WHO report, health care centers unnecessarily complicate the process of hospitalization to the extent that it becomes too exhausting and may even lead the elderly patients to discontinue treatment [43]. Previous studies have also indicated that the treatment process is not tailored to provide optimal care, and the cumbersome hospital admission process causes the frustration of the elderly [31, 44]. Long waiting lists and delays in the provision of treatment were also viewed as a main bottleneck of the health care system [43]. Evidently, the length of hospital stay of elderly patients is longer than in other age groups, particularly in emergency wards, and the incidence of delayed hospital discharges was mainly associated with this age group [45]. Clearly, prolonged hospital stay would negatively affect the treatment outcome of the elderly and may even increase their care needs [46].

Financial abuse was identified as a sub-category of macro-level issues, which was mainly related to organizational policies and structural problems. Elderly patients are more susceptible to financial abuse since they need to repeatedly visit hospitals, require a longer hospital stay, need longer and multiple diagnostic tests, and use more medications. These would certainly increase the financial burden on the elderly to the extent that they may even opt to avoid or delay hospitalization [43]. Our results highlighted various forms of financial abuse; the most odious kind was that of informal payments to health care personnel for favoritism or under-the-table payments to some physicians. A study claimed that informal payment to health care providers is common in many countries. Negative consequences of informal payments were reported as the increased cost of health care, reduced quality of care, tendency to perform unnecessary procedures, and damaging the relationship between the patient and physician/nurse [47].

Another sub-category of macro-level issues was limited resources. In line with our findings, various studies have indicated issues related to the inadequate physical environment of hospitals, shortage of time due to the poor planning, and lack of financial resources as the main bottlenecks to fulfill the essential care needs of elderly patients [44, 48]. Limited resources may lead to a shortage of staff (workforce or lack of time) for the provision of health care tasks (sanitary, nutrition) and behavior (empathy). Such limitations can implicitly shape the mindset of the personnel about patients [31]. Institutional abuse experienced by the hospital personnel (e.g., heavy workload, long working hours, and inadequate equipment or facilities) would negatively affect their behavior and may even endanger their health. As a direct result, they may expose the patients to their anger and frustration [49].

### Limitations

The main limitations of the present study were related to the participants and the boundaries of qualitative research. We felt that our patients were reluctant to clearly express their views on elder abuse; possibly due to the expected backlash from the staff responsible for their treatment and care. This could have undermined a full description of elderly abuse. In addition, since the small sample size was not fully representative of the study population, the findings of our study cannot be generalized to other populations and cultures. Another limitation of this study was the lack of a specific protocol to identify elder abuse at institutions (e.g., hospitals). It underscores the need to develop tools and protocols for the identification, reporting, and management of this type of abuse. However, the main strength of the present qualitative study outweighs its limitations. As a comprehensive study on hospitalized elder abuse, our findings provide useful areas for future research. Inclusion of non-educational hospitals is recommended for future studies. Though in this study, participants’ experiences of abuse could have been influenced by previous hospitalizations in other hospitals.

## Conclusion

The main categories of hospitalized elder abuse were classified as micro-level, meso-level, exo-level, and macro-level issues. The abuse of hospitalized elderly is a multi-factorial and multi-dimensional phenomenon. In addition to individual and professional factors, issues related to the inadequate physical environment and organizational structure of hospitals have drastically contributed to the occurrence of elder abuse. It is recommended that each of these dimensions receives more attention and further extensive and comprehensive evaluations should be conducted to clarify and enrich the existing information.

## Data Availability

The datasets of the current study are not publicly available due to the confidentiality of participants’ data. However, they are available upon reasonable request**.**

## Abbreviations

CCU: Coronary care unit
ICU: Intensive care unit
LOS: Length of stay in hospital
PFM: Patients’ family member
